# Retrospective Study on CO_2_ Laser for Second‐Line Treatment of Vulvar Lichen Sclerosus

**DOI:** 10.1111/jog.70098

**Published:** 2025-09-30

**Authors:** Gian Piero Siliquini, Margherita Giorgi, Concetta Strano, Francesca Accomasso, Beatrice Marina Pennati, Irene Fusco, Tiziano Zingoni, Fabrizio Bert, Valentina Elisabetta Bounous

**Affiliations:** ^1^ Gynecology and Obstetrics Unit Sedes Sapientiae Institute Turin Italy; ^2^ Gynecology and Obstetrics Unit, Mauriziano Umberto I Hospital, Department of Surgical Sciences University of Turin Turin Italy; ^3^ El.En. Group, Clinical Research & Practice Department Calenzano Italy; ^4^ Department of Public Health and Pediatric Sciences University of Turin Turin Italy

**Keywords:** CO_2_ laser, Vulvar Lichen Sclerosus, vulvovaginal atrophy, women's quality of life

## Abstract

**Background:**

Vulvar Lichen Sclerosus (VLS) represents a persistent inflammatory disease of the skin that particularly affects the genital area.

**Aim:**

The study purpose was to investigate the effectiveness of CO_2_ laser as a treatment of recalcitrant VLS. The parameters examined included treatment tolerance, patient satisfaction, symptoms, and VLS clinical markers.

**Methods:**

This retrospective study enrolled 85 adult women affected by Vulvar Lichen Sclerosus (VLS), refractory to local corticosteroids. Patients were treated with three sessions of fractional CO_2_ laser treatment at 4‐week intervals. For vulvovaginal treatment, a device with a scanning handpiece and dedicated probes was used. The effectiveness of the treatment was evaluated at 1 month follow up (T1) and at 6 months follow up (T2) from the last treatment session. The Vulvar Health Index (VuHI) and the Vaginal Health Index score (VHI) were used. Patients assessed the severity of their symptoms (dyspareunia and dryness specifically) using the Visual Analogue Scale (VAS), a 10‐point scale going from 0 (no symptoms) to 10 (maximum symptoms).

**Results:**

VuHI showed significant improvement after completion of treatment compared to baseline (*p* < 0.001). VHI showed significant improvement after completion of treatment compared to baseline (*p* < 0.001). There was clear improvement, compared to baseline values, in the VAS score for superficial dyspareunia and for vulvar dryness.

**Conclusion:**

Overall, the results of this study demonstrated that treatment of recalcitrant VLS with fractional CO_2_ laser improved both clinical assessment and symptoms.

## 1 Introduction

Vulvar Lichen Sclerosus (VLS) represents a persistent inflammatory disease of the skin that particularly affects the genital area. It concerns women at any age, but in pre‐menarchal and postmenopausal periods, it is more frequent [1]. It rarely affects the vaginal mucosa, while the internal section of the labia majora and labia minora, the vestibule, the perineum, the perianal region, and the clitoris are the main areas affected [2]. The most prevalent clinical characteristics are epidermal wrinkles and white‐colored, waxy plaques or papules. Ulcerations and bruises usually appear later during the course of the disease and subsequent scarring [3]. Anatomical changes due to VLS include phimosis of the clitoris, resorption of the labia minora, and narrowing of the vaginal introitus [4]. There may also be hyperpigmentation or depigmentation of the skin. The most prevalent symptoms include dysuria, burning, dyspareunia, persistent vulvar pain, and itching, which is frequently unbearable and typically occurs in the evening and at night [5]. Additionally, the scarring of the foreskin impairs sexual function and Quality of Life (QoL). In addition, it creates distress for the couple, endangers their psychological health, and eventually leads to secondary anorgasmia or apareunia because of the marked decline in vulvar trophism and clitoris sensitivity. Persistent long‐term VLS has also been linked to an elevated risk of squamous cell vulvar carcinoma, which occurs in approximately 3%–9% of cases, especially in patients who do not receive appropriate treatments or have poor compliance due to the side effects of topical treatments prescribed [6, 7]. On the contrary, patients who receive adequate treatments could present symptom resolution, skin re‐pigmentation, and a return to normal skin texture, and are less likely to develop squamous cell carcinoma [8, 9, 10]. Topical ultrapotent steroids are currently the gold‐standard treatment for VLS to reduce symptoms and postpone relapses. Clobetasol propionate is a strong topical steroid that has long been regarded as the gold standard treatment for VLS, with documented efficacy with improvement of the symptoms of 66%–96% [7] and full remission of 23%–54%. It works through anti‐inflammatory, antimitotic, and immunosuppressive effects [7]. Thus, to minimize symptoms and problems, VLS must be diagnosed and treated as soon as possible. Subcutaneous injection of steroids has been shown to be a feasible substitute for topical steroids, with the advantage of injecting the drug directly into the dermis and precisely determining the amount of medication supplied to the target region. On the other hand, long‐term use of high‐potency topical steroids tends to exacerbate skin atrophy, aggravating symptoms of lichen, causing contact sensitization, and raising the risk of secondary infection [11]. Furthermore, in cases of severe disease, resistance to therapy typically develops and shows up as hyperkeratosis and exacerbation of symptoms. Ultrapotent corticosteroid medication might not work in some situations. Numerous substitute treatment approaches have been put out recently [12, 13]. Of these, fractional carbon dioxide (CO_2_) lasers appear to be a viable and effective VLS treatment. The controlled CO_2_ laser energy administration has been shown to improve the sclerosis and atrophy characteristic of VLS by stimulating neoangiogenesis, inducing the creation of glycogen, and increasing collagen production in the lamina propria [14, 15, 16].

Indeed, with a wavelength of 10 600 nm, this kind of laser can cause superficial microablative effects in soft tissues and can also provide a pulsed beam that shields tissues from potential overheating damage. Partial delivery of the laser beam to the tissue results in tiny patches (150–200 μm) that alternate between treated and untreated tissue [17]. Through the creation of the heat shock protein 47, the microablation promotes the remodeling of the connective tissue and results in the production of new collagen, fibroblasts, and ground matrix [18].

The randomized controlled trial by Burkett et al. [19] compared CO_2_ laser with clobetasol propionate. Patients underwent 3 laser treatments, 4–6 weeks apart, with subsequent follow‐up at 6 months. Significant results were obtained, compared to the group treated with clobetasol propionate, regarding the presence of labial phimosis and vulvar erosions (assessed by the objective VAS scale).

In the randomized clinical trial conducted by Salgado et al. [20] that compared CO_2_ laser with propionate clobetasol evaluating the impact on QoL, no significant differences were appreciated between the two treatments. However, satisfaction was greater in the laser group. Probably the higher degree of satisfaction with the fractional CO_2_ laser treatment is due to the convenience of performing the treatment, not requiring daily applications of medication and manipulation of the vulva.

Based on this research, the current study aims to evaluate the performance of CO_2_ fractional laser in the treatment of vulvar VLS refractory to steroid treatments by assessing changes in perceived superficial dyspareunia, vulvar dryness, and vulvar anatomy at 1 and 6 months after the last laser treatment session compared to baseline.

## 2 Material and Methods

### 2.1 Study Population

This retrospective study enrolled 85 women affected by VLS, after failure of local corticosteroid therapy, at the Sedes Sapientiae Institute of Turin in the period between January 2014 and April 2023. Information was collected using an electronic database regarding VLS severity and clinical features, demographic data, medical history, previous treatments, current therapies, and presence of comorbidities. The efficacy of the treatment was evaluated at 1 month follow‐up (T1) and at 6 months follow‐up (T2) from the last treatment session.

Eligibility criteria were: clinical diagnosis of VLS, negative cervical smear in the 12 months before the study, previous medical treatment failure, and signing of an informed consent.

Among the exclusion criteria were: Women with less than 18 years of age; the presence of infection, lesion, or abscess of the genital tract; squamous cell carcinoma; pregnancy or breastfeeding; active urinary infection; vulvar and/or vaginal neoplasia; use of systemic or topical drugs during the study period or in the 2 months prior to the study period; contraindications to the use of laser (such as bacterial, fungal, or active viral infections); alcohol or drug addiction; decompensated psychiatric disorders; unsigned informed consent; or vulvar disease with unclear diagnosis.

Most (94%) of enrolled patients were postmenopausal and had symptoms of vulvo‐vaginal atrophy in addition to those of VLS.

### 2.2 Study Protocol

Patients were treated with three sessions of fractional CO_2_ laser treatment at 4‐week intervals. For vulvovaginal treatment, a device with a scanning handpiece and dedicated probes (Glide, DEKA M.E.L.A., Florence, Italy) was utilized. All procedures were performed by the same operator. In detail, before the scanner moves to the next zone, many emissions are repeated on the same microablation zone (Dermal Optical Thermolysis, DOT). The spacing is the separation between two adjacent microzones (DOTs), while the amount of time the laser emission is applied over each thermal damage (DOT) microzone before moving on to the next is the dwell time.

Laser settings are reported in Table 1. They were selected on the basis of the target area and the extent of the symptoms. For the vaginal canal treatment, parameters are as follows: Power 40 W, dwell time 1000 μs, spacing 1000 μm, smart stack 3. For the vaginal introitus treatment: Power 10–40 W, dwell time 1000 μs, smart stack 1 or 2, spacing 1000 μm. For the vulva, including the clitoris and perianal area: Power 10–40 W, dwell time 1000 μs, smart stack 1 or 2, spacing 1000 μm.

**TABLE 1:** Laser therapy parameters.

Genital area	Probe	Parameters
Vaginal canal	Endo	PulseShape: DP Power: 40 W Dwell time: 1000 μs Spacing: 1000 μm Stack: 3
Vaginal introitus	Vulvar	PulseShape: DP Power: 10–40 W Dwell time: 1000 μs Spacing: 1000 μm Stack: 1–2
Vulva (also clitoris and perianal area)	Vulvar	PulseShape: DP Power: 10–40 W Dwell time: 1000 μs Spacing: 1000 μm Stack: 1–2

**FIGURE 1:**
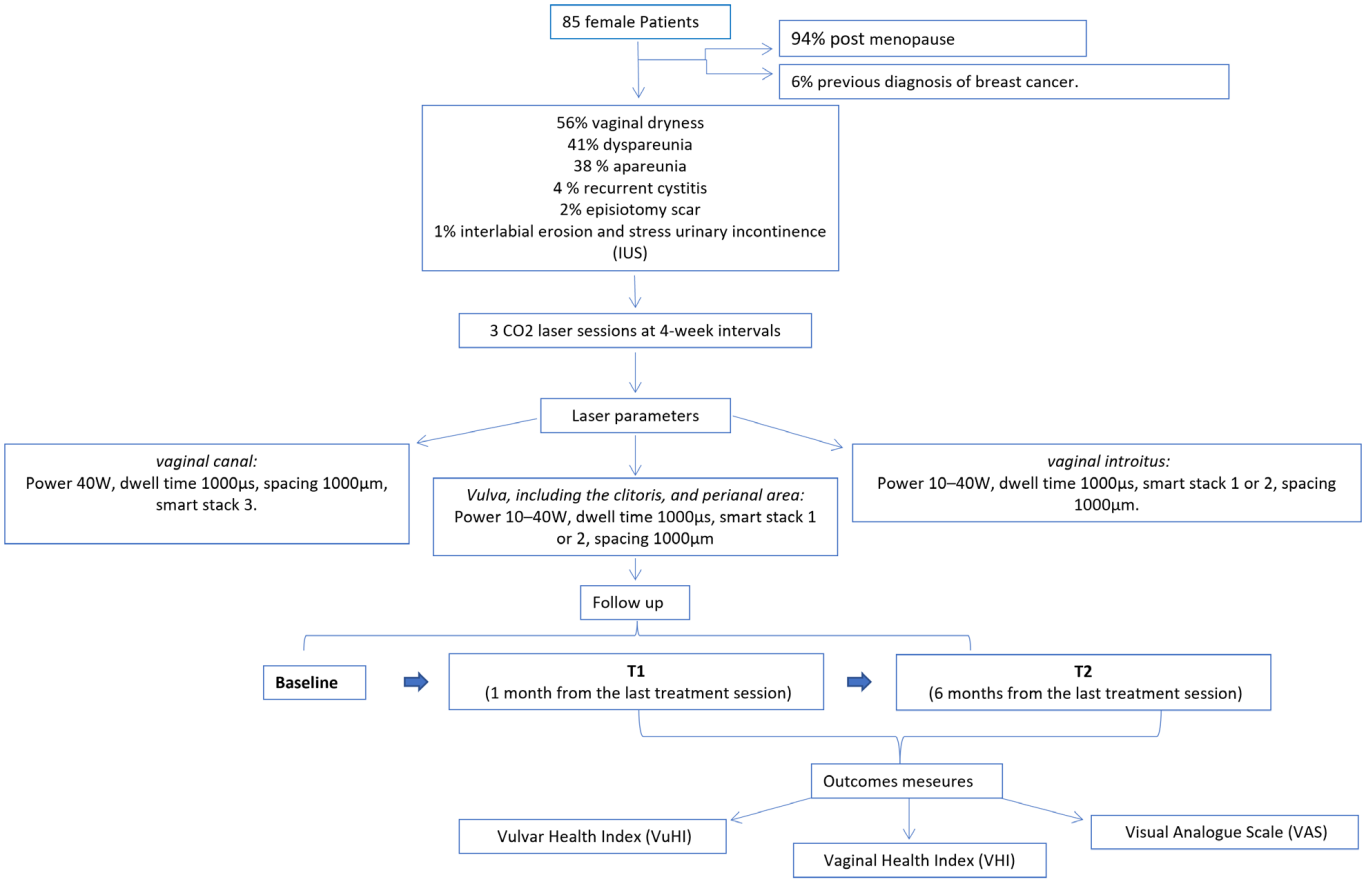
Treatment flowchart.

Every patient was assessed for vulvo‐vaginal and vaginal health at baseline (pre‐treatment), at 1 month from the last treatment session (T1), and 6 months from the last treatment session (T2). The Vulvar Health Index (VuHI) score and the Vaginal Health Index (VHI) score were used. Moreover, patients assessed the severity of their symptoms (superficial dyspareunia and vulvar dryness) using the Visual Analogue Scale (VAS), a 10‐point scale going from 0 (no symptoms) to 10 (maximum symptoms). A flowchart of the treatment is illustrated in Figure 1.

In detail, the Vulvar Health Index (VuHI) consists of eight measures: Labia minora, labia majora, urethra, clitoris, vaginal introitus and elasticity, bother, color and pain, and other elements (such as petechiae and ulcerations). Each parameter goes from 0 to 3. The vulva is deemed atrophic if the overall score is greater than 8 or if the sum for any one of the categories is 3 [21].

On the other hand, the Vaginal Health Index score (VHI) consists of five measures: Elasticity, fluid volume, pH, epithelial integrity, and moisture. Each parameter is graded from 1 to 5. If the total score is < 15, the vagina is considered atrophic [22].

Moreover, patients were asked by a trained interviewer to assess the severity of their symptoms (superficial dyspareunia and vulvar dryness) using the Visual Analogue Scale (VAS), a 10‐point scale going from 0 (no symptoms) to 10 (maximum symptoms) [23].

### 2.3 Statistical Analysis

Statistical analysis was performed using the software SPSS program version 25.0 (IBM).

Initially, the entire sample was subjected to a descriptive analysis, wherein means, interquartile ranges, standard deviations, frequencies, and percentages for categorical variables were reported. Tables were used to show the trends of the mean values of the VAS, VHI, and VuHI scales, which were employed to evaluate the sample.

The outcome's variables examined were: Visual Analogical Scale score in the two items “superficial dyspareunia” and “vulvar dryness”; Vulvar Health Index (VuHI) score and Vaginal Health Index (VHI) score.

A *p* value of less than 0.05 was used to indicate agreement at the level of statistical significance (*T*‐Student test) for all descriptive analyses.

## 3 Results

### 3.1 Study Population

A total of 85 females were enrolled. At baseline, the mean age was 63.7 ± 7.8 years (range, 50–92 years). Most subjects had deliveries (95%), and these were mostly vaginal (54%). Most subjects were postmenopausal (94%). Five patients (6%) had a previous diagnosis of breast cancer.

Among declared symptoms, the most common was vaginal dryness (56%), followed by dyspareunia (*n* 41%), apareunia (38%), recurrent cystitis (4%), episiotomy scar (2%), interlabial erosion, and stress urinary incontinence (IUS, 1%) (Table 2).

**TABLE 2:** Characteristics of the study population.

*N* = 85	Range	Mean ± SD	Median
Age (years)	50–92	63.7 ± 7.8	63
High (cm)	152–180	163.2 ± 5.8	162
Weight (kg)	42–105	62.0 ± 10.4	61
BMI	17.1–37.5	23.2 ± 3.6	22.3
Smoking habits			
Smokers	13 (15%)		
Ex‐smokers	8 (9%)		
Smoking period (years)	5–40	20.4 ± 12.7	20
Smoked cigarettes/day	3–40	17.3 ± 10.3	20
Previous breast cancer	5 (6%)		
Menopause	82 (94%)		
Age of menopause (years)	30–57	50.2 ± 4.6	51
Symptoms	85 (100%)		
Apareunia	32 (38%)		
Vaginal dryness	48 (56%)		
Dyspareunia	35 (41%)		
Recurrent cystitis	3 (4%)		
Interlabial erosion	1 (1%)		
SUI	1 (1%)		

Abbreviations: BMI, body mass index; SD, standard deviation; SUI, stress urinary incontinence.

### 3.2 VuHI Evaluation

Results of the VuHI are reported in Table 3 (see Supporting Information S1). The eight measures regarding the labia minora, labia majora, urethra, clitoris, vaginal introitus, elasticity, bother and pain, color, and other elements (such as petechiae and ulcerations) of the vulvovaginal area showed a significant (*p* < 0.001) improvement after completion of treatment compared to baseline.

**TABLE 3:** The Vulvar Health Index (VuHI—average ± standard deviation at T0, T1, and T2).

VuHI	T0	T1	T2	*p*
Labia majora	1.1 ± 0.8	0.6 ± 0.6	0.4 ± 0.5	*p* < 0.001
Labia minora	2.1 ± 1.0	1.6 ± 1.2	1.6 ± 1.2	*p* < 0.001
Clitoris	2.0 ± 1.0	1.6 ± 1.2	1.5 ± 1.2	*p* < 0.001
Urethra	0.2 ± 0.5	0.1 ± 0.2	0.0 ± 0.0	*p* < 0.001
Vaginal introitus and elasticity	1.3 ± 1.0	0.8 ± 0.8	0.6 ± 0.7	*p* < 0.001
Color	2.4 ± 0.8	1.5 ± 0.9	1.1 ± 0.8	*p* < 0.001
Bother and pain	2.4 ± 0.9	1.8 ± 1.1	1.0 ± 1.0	*p* < 0.001
Other elements (e.g., ulcerations, petechiae)	2.1 ± 1.1	1.7 ± 1.1	1.4 ± 1.1	*p* < 0.001
Total	13.6 ± 3.7	9.7 ± 4.4	7.5 ± 4.0	*p* < 0.001

### 3.3 VHI Evaluation

Results of the VHI are reported in Table 4 (see Supporting Information S2). The five measures regarding elasticity, fluid secretion, pH, epithelial mucosa, and moisture of the vaginal area showed significant (*p* < 0.001) improvement after completion of treatment compared to baseline.

**TABLE 4:** The Vaginal Health Index score (VHI—average ± standard deviation at T0, T1, and T2).

VHI	T0	T1	T2	*p*
Elasticity	3.4 ± 1.0	4.3 ± 0.8	4.5 ± 0.7	*p* < 0.001
Fluid secretion	2.6 ± 1.3	3.8 ± 1.0	4.0 ± 1.1	*p* < 0.001
pH	1.8 ± 1.1	2.0 ± 1.3	2.2 ± 1.4	*p* < 0.001
Epithelial mucosa	3.6 ± 0.8	3.8 ± 0.5	4.0 ± 0.4	*p* < 0.001
Moisture	3.3 ± 0.9	4.1 ± 0.9	4.4 ± 0.9	*p* < 0.001
Total	14.7 ± 3.9	18.0 ± 3.1	19.1 ± 2.8	*p* < 0.001

### 3.4 VAS Evaluation

VAS scores are reported in Table 5 (see Supporting Information S3). There was significant (*p* < 0.001) improvement compared to baseline values in the VAS score for superficial dyspareunia and in the VAS score for vulvar dryness.

**TABLE 5:** The Visual Analogue Scale (VAS): Average ± standard deviation at T0, T1, and T2.

Symptom	T0	T1	T2	*p*
Superficial dyspareunia	5.6 ± 3.8	2.6 ± 2.9	2.0 ± 2.5	*p* < 0.001
Vulvar dryness	7.1 ± 2.6	4.6 ± 2.7	2.5 ± 2.5	*p* < 0.001

Most patients reported vaginal, vulvar, and introitus laser treatment as pain‐free.

No side effects were observed.

## 4 Discussion

VLS is a chronic inflammatory illness that is often underdiagnosed and challenging to treat. Currently, the first‐line therapy involves topical corticosteroids. However, these have limitations due to potential long‐term side effects and often poor response, as VLS frequently presents as resistant to medical therapies [24].

New horizons in regenerative therapy for VLS are being explored such as laser, vulvar lipofilling, and platelet‐rich plasma injection. Laser therapy in the gynecological field has shown significant results, particularly in women with genitourinary syndrome of menopause (GSM) [25].

Consequently, vulvar laser therapy has been considered a treatment option in recent studies. Although current research is limited and conducted on small sample sizes, fractional CO_2_ laser therapy has been suggested by several trials and case series as a potentially effective treatment for VLS [26, 27, 28, 29, 30].

Especially in steroid‐resistant cases, promising results seem to come from regenerative therapies, including CO_2_ laser treatment [25].

Concerning the mechanism of action of CO_2_ laser, according to literature [26, 27, 28, 29, 30], treating VLS with fractional CO_2_ laser therapy may promote protein synthesis, accelerate tissue regeneration, and reduce lichenification of the skin. There also appears to be improvement in both clinical signs and symptoms, including severe vulvar itching and burning. Furthermore, Marzec et al. [31] demonstrated that CO_2_ laser treatment can alter the expression of genes related to collagen, elastin, heat shock protein, and p53.

In our study after 3 sessions of treatment with laser, the color, moisture, and elasticity of the mucosa and skin improved considerably, and nearly all women reported that the laser treatment was well tolerated and enhanced their QoL. The effect of the laser on vulvar health was evaluated, measured by objective (VuHI scale) and subjective instruments, using validated scales (VAS scale for superficial dyspareunia and vulvar dryness). Both the VuHI score and the VAS scale showed significant improvement in the assessed parameters at 1 and at 6 months after the laser treatment was completed. A significant result was also obtained with laser treatment at the vaginal level for GSM symptoms, which was evaluated by the VHI score.

Specifically, for the VuHI, the eight measures regarding the labia minora, labia majora, urethra, clitoris, vaginal introitus, elasticity, bother and pain, color, and other elements (such as petechiae and ulcerations) of the vulvovaginal area showed a significant (*p* < 0.001) improvement after completion of treatment compared to baseline.

When compared to baseline, the VHI evaluation's five metrics—elasticity, fluid secretion, pH, epithelial mucosa, and vaginal region moisture—showed a significant (*p* < 0.001) improvement following therapy.

Finally, VAS scores significantly (*p* < 0.001) improved, compared to baseline values.

Our study showed that laser therapy may be a promising therapeutic alternative for VLS patients refractory to medical therapies, leading to improvement in vulvar health objectively assessed and in VLS‐related symptoms subjectively assessed by validated instruments.

Our results are in line with Filippini et al. [32] who describe that fractional CO_2_ laser represents a successful alternative treatment with no significant discomfort or side effects. It considerably reduced all the symptoms that patients described, including vaginal introitus discomfort, vaginal dryness, dyspareunia, burning, and itching. In addition, they noted that in many of their patients also presenting GSM, good improvement in both conditions was reported as a further benefit and validation of the treatment's efficacy.

Significant improvement in symptoms assessed by the subjective VAS scale was also highlighted by the study of Pagano et al. [25], a prospective longitudinal study, in which 2 cycles of CO_2_ laser therapy were performed with an interval of 30–40 days, subsequently implementing a follow‐up at a distance of 5 months. Symptoms evaluated, in addition to dyspareunia and dryness, included itching, vulvodynia, and reduced sensitivity during intercourse. In agreement with our study, dyspareunia was found to improve significantly.

The efficacy of CO_2_ laser therapy was also assessed by the group of Krause et al. [33], who treated 67 VLS patients with the CO_2_ laser, comparing two different powers; they observed a significant improvement in VAS score in both groups. In line with our setting, laser parameters, the irradiation of the normal dose group was adjusted with a power of 24 W, an exposure time of 400 μs, and a spacing of 1000 DOT, while the low dose group received a lower dose of 0.5 W power with the same exposure time and spacing. Surprisingly, the lowest dose that was supposed to work as a placebo was found to be effective.

Additionally, the findings of Villalba et colleagues [34] highlight the potential of fractional CO_2_ laser therapy as a safe and effective adjunct for refractory LS. In this prospective observational study, the authors demonstrated significant reductions in key symptoms, including pruritus, pain, and dyspareunia, with an improvement in patients' quality of life and sexual function without adverse effects.

Overall, in accordance with the literature's findings, our study showed that treating recalcitrant VLS with fractionated CO_2_ laser can improve both clinical assessment and symptoms.

In our opinion, this regenerative technique should be implemented, given the excellent results and the limited therapeutic alternatives currently available.

### 4.1 Study Limitation

Within the limitations of this study, there is a lack of younger patients in the study population. It would be more complete and more interesting to understand if the treatment we used is as efficient as it was in older women. Other biases are the absence of a non‐randomized design, a control group, the lack of long‐term follow‐up, and most importantly, the potential to produce a placebo effect, even though the number of patients treated was representative.

## Author Contributions

**Gian Piero Siliquini:** conceptualization, data curation, formal analysis, funding acquisition, investigation, methodology, project administration, resources, software, supervision, validation, visualization, writing – original draft, writing – review and editing. **Margherita Giorgi:** conceptualization, data curation, formal analysis, investigation, methodology, resources, software, supervision, validation, visualization, writing – original draft, writing – review and editing. **Concetta Strano:** conceptualization, data curation, formal analysis, investigation, methodology, resources, software, supervision, validation, visualization, writing – original draft, writing – review and editing. **Francesca Accomasso:** conceptualization, data curation, formal analysis, investigation, methodology, resources, software, supervision, validation, visualization, writing – original draft, writing – review and editing. **Beatrice Marina Pennati:** visualization, writing – original draft, writing – review and editing. **Irene Fusco:** visualization, writing – original draft, writing – review and editing. **Tiziano Zingoni:** funding acquisition, project administration, supervision, validation, visualization, writing – original draft, writing – review and editing. **Fabrizio Bert:** conceptualization, data curation, formal analysis, investigation, methodology, resources, software, supervision, validation, visualization, writing – original draft, writing – review and editing. **Valentina Elisabetta Bounous:** conceptualization, data curation, formal analysis, investigation, methodology, resources, software, supervision, validation, visualization, writing – original draft, writing – review and editing.

## Disclosure

The article has not been presented in abstract form at a conference or as part of a presentation.

## Ethics Statement

The article is in accordance with the principles of the Declaration of Helsinki on Ethical Principles for Medical Research involving human subjects. Ethical approval was not required as the study devices have already been CE marked since 2021. No activity was carried out outside the scope of the device's intended purpose or that no additional invasive or burdensome procedures were carried out compared to procedures performed under the normal condition of use of the device.

## Consent

Informed consent was received from all participants.

## Conflicts of Interest

B.M.P., I.F., and T.Z. are employed at El.En. Group. The other authors declare no conflicts of interest.

## Supporting information


**Supporting Information S1:** Raw data of VuHI scores.

**Supporting Information S2:** Raw data of VHI scores.

**Supporting Information S3:** Raw data of VAS scores.

## Data Availability

The data that supports the findings of this study is available in the Supporting Information of this article.
