# Supplementary material for: Retrospective Study on CO2 Laser for Second‐Line Treatment of Vulvar Lichen Sclerosus
Source: J Obstet Gynaecol Res. 2025 Sep 30;51(10):e70098. doi: 10.1111/jog.70098 (PMC12484719; doi:10.1111/jog.70098)
Supplement: Supplementary file 1 — Supporting Information S1: Raw data of VuHI scores. [file JOG-51-0-s002.pdf]

T0	PLMaj	PLMin	PClito	PUre	PIntro	PColo	Ppain	Pother	TOTAL	T1	PLMaj	PLMin	PClito	PUre	PIntro	PColo	Ppain	Pother	TOTAL	T2	PLMaj	PLMin	PClito	PUre	PIntro	PColo	Ppain	Pother	TOTAL
	3	2	2	1	0	2	3	0	13		1	1	2	0	1	1	1	1	8		1	1	0	0	1	1	1	0	5
	2	2	2	2	2	3	3	0	16		0	0	0	0	1	2	1	0	4		0	1	2	0	1	1	1	0	6
	3	3	1	2	3	3	3	0	18		1	2	1	1	1	1	2	0	9		0	1	1	0	1	1	0	0	4
	3	3	2	0	2	3	2	2	17		1	2	1	0	0	1	0	0	5		0	0	1	0	0	1	0	0	2
	1	1	0	0	1	3	2	3	11		1	1	0	0	0	2	2	3	9		1	1	0	0	0	1	2	3	8
	1	1	1	1	2	2	3	3	14		0	0	0	1	0	1	0	1	3		0	1	0	0	0	1	2	0	4
	1	1	1	0	1	2	3	0	9		1	0	1	0	1	1	2	0	6		1	1	1	0	1	2	3	0	9
	1	1	0	0	2	2	3	0	9		0	0	0	0	1	2	3	0	6		0	0	0	0	1	2	3	0	6
	2	1	2	0	1	3	1	0	10		2	0	0	0	0	0	0	0	2		0	0	0	0	0	1	0	0	1
	1	1	1	1	1	3	2	2	12		0	0	0	0	0	1	1	2	4		0	0	1	0	0	1	0	2	4
	2	2	2	0	1	3	2	0	12		2	1	2	0	1	1	0	0	7		1	1	0	0	1	0	0	0	3
	1	1	1	0	1	1	2	3	10		0	1	0	0	0	1	0	0	2		0	1	1	0	0	1	1	0	4
	1	0	1	0	1	1	2	0	6		1	0	0	0	1	1	1	0	4		0	0	0	0	1	0	0	0	1
	2	3	3	0	1	3	3	2	17		1	3	3	0	1	1	2	2	13		1	3	3	0	1	1	1	2	12
	1	3	3	0	3	3	3	3	19		1	3	3	0	3	3	3	3	19		1	3	2	0	3	3	3	3	18
	1	3	3	0	1	2	2	0	12		0	0	0	0	1	1	3	1	6		0	0	0	0	0	0	1	0	1
	1	1	1	0	2	2	3	1	11		0	0	0	1	0	1	1	0	3		0	0	0	0	1	0	1	0	2
	2	2	3	0	3	3	3	3	19		2	1	1	0	1	2	3	1	11		1	0	1	0	1	2	2	2	9
	0	2	2	0	0	2	1	3	10		0	0	0	0	0	0	0	1	1		0	1	1	0	0	0	0	0	2
	1	2	2	0	2	2	3	3	15		1	2	2	0	1	1	2	3	12		0	3	3	0	1	1	0	3	11
	1	3	3	0	1	3	3	3	17		1	3	3	0	1	2	2	3	15		0	3	3	0	0	1	0	2	9
	1	1	1	0	2	3	3	3	14		1	0	0	0	1	1	3	2	8		1	0	0	0	1	1	3	2	8
	2	3	3	0	2	2	2	3	17		1	3	2	0	2	1	1	3	13		1	3	2	0	2	1	1	3	13
	1	1	3	0	2	2	2	2	13		1	1	3	0	2	1	1	2	11		1	2	2	0	0	1	0	2	8
	1	1	2	1	1	2	3	1	12		1	1	1	1	1	1	3	1	10		1	1	0	0	1	1	2	0	6
	1	3	3	0	1	3	2	3	16		1	3	3	0	1	3	2	3	16		1	3	3	0	1	3	2	3	16
	1	1	1	0	2	2	3	2	12		0	0	1	0	2	1	3	2	9		0	1	3	0	1	1	1	2	9
	1	2	2	0	1	2	3	1	12		1	1	0	0	0	1	1	1	5		0	1	0	0	0	0	1	1	3
	2	2	3	0	1	2	3	3	16		2	2	3	0	1	2	3	2	15		0	2	2	0	1	1	1	2	9
	0	0	0	0	0	3	0	2	5		0	0	0	0	0	3	0	2	5		0	0	0	0	0	2	0	1	3
	0	3	3	0	1	1	3	2	13		0	2	3	0	0	0	1	1	7		0	2	3	0	0	0	0	1	6
	1	1	1	1	1	1	3	1	10		0	1	1	0	0	1	2	0	5		0	2	0	0	0	1	0	1	4
	1	2	3	0	2	3	3	2	16		1	1	3	0	1	1	3	1	11		0	1	1	0	1	1	3	1	8
	1	3	3	0	1	3	3	3	17		0	2	3	0	0	2	2	3	12		0	3	3	0	0	1	0	1	8
	1	1	0	2	1	2	3	2	12		0	0	0	0	0	2	1	3	6		1	0	0	0	0	2	0	1	4
	0	3	3	0	3	3	3	3	18		0	3	3	0	3	2	3	3	17		0	3	3	0	3	2	3	3	17
	1	1	2	0	0	3	0	2	9		1	0	2	0	0	3	0	2	8		1	0	1	0	0	2	0	2	6
	2	3	3	0	3	3	3	3	20		1	3	3	0	2	2	2	3	16		1	3	3	0	2	1	1	3	14
	1	3	2	0	0	2	3	3	14		1	3	2	0	0	0	3	3	12		0	3	1	0	0	0	3	3	10
	1	3	2	0	1	3	2	3	15		1	3	1	0	1	3	2	3	14		1	3	1	0	1	3	2	3	14
	1	3	3	0	1	3	3	3	17		0	1	0	0	1	0	0	1	3		0	0	0	0	0	0	0	0	0
	1	3	1	0	2	3	3	3	16		0	2	1	0	1	2	3	3	12		0	1	0	0	1	0	0	1	3
	1	3	3	0	2	2	3	3	17		0	3	2	0	1	1	3	3	13		0	3	1	0	1	1	1	2	9
	1	3	3	0	0	3	3	3	16		1	3	3	0	0	1	0	3	11		1	3	3	0	0	1	0	3	11
	1	3	3	0	1	2	3	3	16		0	3	3	0	0	1	3	3	13		0	3	3	0	0	1	1	3	11
	0	0	0	0	1	0	3	1	5		0	0	0	0	1	0	2	1	4		0	0	0	0	1	0	2	1	4
	0	2	3	1	2	3	3	2	16		0	2	3	0	2	2	3	1	13		0	3	1	0	1	1	2	0	8
	0	2	1	0	2	2	3	0	10		0	2	1	0	1	1	3	0	8		0	1	1	0	1	1	1	0	5
	0	1	1	0	0	1	2	0	5		0	1	1	0	0	1	2	0	5		0	1	1	0	0	1	2	0	5
	1	2	1	0	1	2	1	2	10		1	1	1	0	0	1	2	2	8		1	1	1	0	0	1	2	2	8
	1	3	3	0	0	3	2	3	15		0	3	3	0	0	2	2	3	13		0	3	3	0	0	2	1	3	12
	0	0	0	0	0	3	3	3	9		0	0	0	0	0	3	2	2	7		0	0	1	0	0	1	1	2	5
	1	2	1	0	2	3	3	1	13		1	2	1	0	1	1	1	1	8		0	2	0	0	1	1	1	1	6
	2	1	1	0	0	2	3	2	11		2	1	1	0	0	1	1	2	8		2	1	1	0	0	1	1	0	6
	1	0	0	0	0	1	3	2	7		0	0	0	0	0	1	3	1	5		0	0	0	0	0	0	3	1	4
	0	3	3	0	0	0	0	3	9		0	3	3	0	0	0	0	1	7		0	3	3	0	0	0	0	0	6
	2	3	3	0	1	3	0	3	15		0	1	2	0	1	1	0	3	8		0	1	2	0	0	1	0	1	5
	2	3	3	0	3	3	3	3	20		1	3	3	0	2	2	3	3	17		1	1	3	0	1	2	0	2	10
	0	3	1	0	1	2	3	2	12		0	3	1	0	1	1	3	2	11		0	1	0	0	0	1	0	1	3
	1	3	3	0	1	3	2	3	16		0	2	3	0	0	1	2	2	10		1	1	3	0	0	1	1	1	8
	0	2	3	0	2	3	2	3	15		0	2	3	0	2	3	2	3	15		0	2	3	0	1	2	0	2	10
	1	3	3	0	2	3	3	2	17		1	3	3	0	1	3	2	2	15		1	3	3	0	0	0	1	2	10
	1	3	3	0	1	3	3	3	17		1	3	3	0	1	2	3	3	16		0	2	3	0	1	1	0	3	10
	1	2	2	0	1	3	3	2	14		1	2	2	0	1	2	3	2	13		0	2	2	0	1	1	3	2	11
	2	3	3	0	0	3	3	3	17		2	3	3	0	0	2	3	3	16		2	3	2	0	0	1	2	0	10
	0	3	2	0	2	3	3	3	16		0	2	2	0	1	2	3	2	12		0	2	2	0	1	2	2	2	11
	1	3	2	0	3	3	1	3	16		1	3	2	0	3	3	0	3	15		0	3	2	0	2	2	0	3	12
	1	3	3	0	0	3	0	2	12		1	3	3	0	0	3	0	2	12		1	3	3	0	0	3	0	2	12
