# Supplementary material for: Retrospective Study on CO2 Laser for Second‐Line Treatment of Vulvar Lichen Sclerosus
Source: J Obstet Gynaecol Res. 2025 Sep 30;51(10):e70098. doi: 10.1111/jog.70098 (PMC12484719; doi:10.1111/jog.70098)
Supplement: Supplementary file 2 — Supporting Information S2: Raw data of VHI scores. [file JOG-51-0-s001.pdf]

T0	Pela	Psecr	PpH	PMuc	Pmois	TOT	T1	Pela	Psecr	PpH	PMuc	Pmois	TOT	T2	Pela	Psecr	PpH	PMuc	Pmois	TOT
	2	1	1	1	1	6		4	4	1	3	3	15		4	4	1	3	3	15
	1	1	1	2	2	7		2	3	1	4	2	12		4	3	3	4	4	18
	1	1	1	1	2	6		3	3	4	3	3	16		3	5	4	4	4	20
	2	2	1	3	2	10		5	4	2	4	4	19		5	5	1	4	5	20
	4	3	1	3	4	15		4	4	1	4	3	16		5	5	1	4	5	20
	1	1	4	1	2	9		4	4	1	3	4	16		4	4	5	3	4	20
	3	2	1	4	3	13		4	4	1	4	4	17		5	4	1	4	5	19
	2	2	5	3	2	14		3	2	5	3	3	16		4	3	5	4	3	19
	4	2	1	4	3	14		5	4	5	4	4	22		5	5	3	5	5	23
	3	2	1	3	3	12		5	4	1	3	5	18		5	4	3	5	5	22
	4	1	1	4	2	12		4	4	1	4	4	17		4	5	1	4	4	18
	5	5	4	4	5	23		5	5	4	4	5	23		5	4	4	4	5	23
	3	2	1	4	4	14		5	4	1	4	4	18		5	5	1	4	5	20
	4	1	3	4	3	15		4	3	3	4	4	18		5	4	3	4	5	21
	3	3	1	4	3	14		4	4	1	4	4	17		4	4	1	4	4	17
	4	2	3	4	3	16		4	4	4	4	4	20		4	4	4	4	4	20
	4	3	4	4	4	19		5	5	4	4	5	23		5	5	4	4	5	23
	3	1	1	3	2	10		4	3	2	4	4	17		4	3	3	4	4	18
	4	4	3	3	4	18		5	5	5	5	5	25		5	5	5	5	5	25
	4	2	3	4	4	17		5	4	2	4	5	20		5	5	2	4	5	21
	4	2	2	3	3	14		5	4	1	4	5	19		5	5	2	4	5	21
	2	1	1	3	2	9		5	3	1	4	4	17		5	3	1	4	4	17
	3	2	1	4	3	13		4	4	1	4	4	17		4	4	1	4	4	17
	4	4	3	4	4	19		4	5	4	4	5	22		5	5	5	4	5	24
	2	2	1	2	2	9		4	4	1	4	4	17		5	5	1	4	5	20
	3	4	1	4	3	15		5	5	1	4	5	20		5	5	1	4	5	20
	4	3	1	3	4	15		4	4	1	3	4	16		4	5	4	5	5	23
	4	3	2	2	3	14		4	5	2	3	5	19		5	1	2	4	5	17
	4	4	1	4	5	18		4	4	1	4	5	18		5	4	1	4	5	19
	4	4	4	4	4	20		5	5	4	4	5	23		5	5	4	4	5	23
	5	5	4	5	5	24		5	5	4	5	5	24		5	5	5	5	5	25
	4	4	4	4	4	20		5	5	4	4	5	23		5	5	5	4	5	24
	4	2	1	3	4	14		5	4	2	3	5	19		5	4	2	4	5	20
	4	1	4	4	5	18		4	4	3	4	5	20		4	4	3	4	5	20
	3	3	2	3	3	14		4	4	3	4	4	19		5	1	4	4	5	19
	4	4	1	4	4	17		4	4	1	3	4	16		4	4	1	3	4	16
	5	5	2	4	5	21		5	5	2	4	5	21		5	5	3	4	5	22
	4	2	1	4	4	15		4	2	1	4	4	15		4	2	1	4	4	15
	4	2	1	3	4	14		5	4	1	3	5	18		5	5	1	3	5	19
	1	1	1	4	2	9		1	1	1	4	2	9		1	1	1	4	3	10
	4	4	2	4	5	19		4	4	2	4	5	19		4	4	2	4	5	19
	4	4	3	4	4	19		5	4	3	4	5	21		5	4	3	4	5	21
	4	1	1	4	3	13		5	3	1	4	4	17		5	4	1	5	5	20
	5	5	1	4	4	19		5	5	1	4	5	20		5	5	1	4	5	20
	4	2	1	4	4	15		5	3	1	4	5	18		5	4	1	4	5	19
	5	5	2	4	5	21		5	5	2	4	5	21		5	5	2	4	5	21
	0	0	0	0	0	0		4	5	1	4	5	19		5	5	1	4	4	19
	2	1	2	4	3	12		4	3	2	4	5	18		5	4	2	4	5	20
	5	4	2	3	2	16		5	4	2	3	2	16		5	4	2	3	2	16
	4	4	1	4	4	17		5	4	1	4	4	18		5	4	1	4	4	18
	4	3	1	4	3	15		5	4	1	4	4	18		5	5	1	4	4	19
	5	5	3	5	5	23		5	5	3	5	5	23		5	1	3	4	5	18
	4	2	1	4	4	15		5	4	1	4	5	19		5	4	1	4	5	19
	4	4	1	4	4	17		4	4	1	4	4	17		4	4	1	4	3	16
	4	4	1	4	4	17		4	5	1	4	4	18		4	5	1	4	4	18
	4	5	3	4	4	20		4	5	3	4	3	19		4	5	3	4	3	19
	4	3	2	4	4	17		4	3	2	4	4	17		4	2	2	4	4	16
	2	3	1	4	3	13		3	3	1	4	3	14		5	3	1	4	5	18
	3	2	1	4	3	13		4	3	1	4	4	16		4	4	1	4	4	17
	4	3	3	4	4	18		5	5	3	4	5	22		5	5	3	4	5	22
	4	1	3	4	3	15		4	3	3	4	4	18		4	3	3	4	4	18
	2	1	1	3	2	9		3	1	1	3	2	10		4	4	1	3	2	14
	3	1	1	4	3	12		4	1	1	4	4	14		4	4	1	4	4	17
	2	3	1	3	2	11		3	3	1	4	3	14		3	3	1	4	3	14
	3	1	1	3	2	10		5	4	1	3	2	15		5	4	1	4	3	17
	2	2	1	4	3	12		4	3	1	4	4	16		4	3	1	4	4	16
	4	2	2	4	4	16		4	3	2	4	4	17		4	3	2	4	4	17
	4	4	4	4	4	20		4	4	4	4	4	20		4	4	4	4	4	20
