# Supplementary material for: Retrospective Study on CO2 Laser for Second‐Line Treatment of Vulvar Lichen Sclerosus
Source: J Obstet Gynaecol Res. 2025 Sep 30;51(10):e70098. doi: 10.1111/jog.70098 (PMC12484719; doi:10.1111/jog.70098)
Supplement: Supplementary file 3 — Supporting Information S3: Raw data of VAS scores. [file JOG-51-0-s003.pdf]

T0	Sup. Dyspareunia	Vulvar Dryness	T1	Sup. Dyspareunia	Vulvar Dryness	T2	Sup. Dyspareunia	Vulvar Dryness
	0	10		0	3		0	2
	9	9		6	4		2	2
	9	7		7	6		2	0
	9	9		4	4		2	1
	10	9		6	1		1	1
	0	5		0	2		0	0
	6	6		3	3		3	3
	10	6		10	3		10	2
	8	6		2	3		1	0
	8	8		5	3		0	2
	0	8		0	3		0	1
	5	5		3	4		4	0
	6	7		2	3		3	3
	10	8		0	6		0	3
	0	8		0	8		0	7
	8	10		3	7		5	7
	2	10		0	6		0	2
	9	10		8	8		0	0
	7	7		0	8		0	1
	2	9		0	7		2	0
	0	10		0	8		0	1
	0	10		0	8		0	6
	7	8		3	1		3	1
	7	7		4	5		0	1
	5	10		0	8		6	0
	6	6		8	4		8	4
	0	10		0	9		0	4
	8	5		4	2		3	2
	7	10		6	10		6	10
	0	2		0	2		0	2
	6	2		5	1		1	0
	8	8		0	7		0	2
	9	9		0	8		9	0
	6	5		0	3		1	1
	10	10		1	1		1	1
	0	0		0	0		0	0
	0	0		0	0		0	0
	3	8		0	7		0	3
	3	9		0	7		1	6
	10	0		3	0		0	0
	7	7		3	7		1	7
	10	5		0	3		0	2
	10	6		1	0		1	0
	0	10		0	4		0	2
	5	10		5	2		5	1
	7	7		3	5		1	10
	10	9		10	7		10	2
	9	9		8	8		6	6
	5	5		3	4		0	0
	3	5		3	5		2	0
	7	6		4	3		4	3
	8	6		5	5		1	0
	8	6		4	6		4	6
	0	7		3	2		1	2
	0	10		0	8		0	8
	2	4		0	3		0	3
	10	7		3	0		3	0
	3	10		0	8		3	2
	10	5		4	2		2	1
	7	7		5	4		3	3
	5	8		0	2		0	2
	7	7		4	4		4	4
	10	10		7	2		4	1
	10	10		3	10		3	7
	10	10		3	6		6	4
	0	5		0	5		0	3
	0	5		0	3		0	3
	0	7		0	7		0	0
